# Remedial Teaching and Learning From a Cognitive Diagnostic Model Perspective: Taking the Data Distribution Characteristics as an Example

**DOI:** 10.3389/fpsyg.2021.628607

**Published:** 2021-03-24

**Authors:** He Ren, Ningning Xu, Yuxiang Lin, Shumei Zhang, Tao Yang

**Affiliations:** ^1^Collaborative Innovation Center of Assessment for Basic Education Quality, Beijing Normal University, Beijing, China; ^2^School of Statistics, Beijing Normal University, Beijing, China; ^3^Department of Statistics, University of California, Davis, Davis, CA, United States

**Keywords:** cognitive diagnostic models, DINA, mathematics teaching, data distribution characteristics, formative assessment

## Abstract

In response to the big data era trend, statistics has become an indispensable part of mathematics education in junior high school. In this study, a pre-test and a post-test were developed for the six attributes (sort, median, average, variance, weighted average, and mode) of the data distribution characteristic. This research then used the cognitive diagnosis model to learn about the poorly mastered attributes and to verify whether cognitive diagnosis can be used for targeted intervention to improve students' abilities effectively. One hundred two eighth graders participated in the experiment and were divided into two groups. Among them, the intervention materials read by the experimental group students only contained attributes that they could not grasp well. In contrast, the reading materials of the control group were non-targeted. The results of the study showed the following: (1) The variance and the weighted average were poorly mastered by students in the pre-test; (2) compared with the control group, the average test score of the experimental group was significantly improved; (3) in terms of attributes, the experimental group students' mastery of variance and the weighted average was significantly improved than the pre-test, while the control group's mastery was not. Based on this, some teaching suggestions were put forward.

## Introduction

For a long time, statistics has been the concern of a few researchers in the field of middle school mathematics education (Burrill, 1990). The research of Garfield and Ahlgren (1988) showed that until the 1980s, students still had few opportunities to learn statistics before entering the university. However, since the beginning of the 21st century, the explosive growth of data accessibility has made understanding and application of statistical literacy essential in all walks of life (Galesic and Garcia-Retamero, 2010; Schield, 2010; Ridgway et al., 2011; Watson, 2014). Indeed, citizens who lack statistical knowledge may not be able to distinguish between credible and unreliable information, and it is difficult for them to make decisions based on data rather than feelings (English and Watson, 2016). For the first time, people see the value of statistical literacy and widely regard it as an essential life skill for fully functional citizens (Ridgway et al., 2011). In response to this trend, statistics has become a focus of concern for many countries and has become an indispensable part of the middle school math curriculum (Lee and Lee, 2008; Arican and Kuzu, 2019). In the United States, the National Council of Teachers of Mathematics [National Council of Teachers of Mathematics (NCTM), 2000] explicitly regarded data analysis and probability as one of the five content standards for the mathematics curriculum. In China, the mathematics curriculum standards included the content of “probability and statistics” as an independent learning module for the first time in 2001 (Ministry of Education of the People's Republic of China, 2001). Furthermore, studies have shown that an essential part of middle schools' statistics education is to teach students to choose appropriate statistical methods to analyze data and extract the information in the data (Franklin et al., 2007). In the *Curriculum Focal Points* [National Council of Teachers of Mathematics (NCTM), 2010], “analyzing and summarizing data sets” is also regarded as one of the key points of middle school math learning. In China's mathematics curriculum standards, it is clearly stated that students should master commonly used methods of measuring the center and variation of data sets in order to carry out an analysis. Therefore, this study will select the data distribution characteristics: the center and variation of the data as the research content.

Although the above statistical knowledge is highly valued, studies have shown that there are often some problems in the learning and teaching of this knowledge (Franklin et al., 2007; Batanero and Díaz, 2012). Suppose we can accurately obtain students' mastery of relevant knowledge and conduct analysis to provide teachers with teaching guidance. In that case, we can help improve these learning and teaching problems and enhance students' ability to solve statistical problems. Nevertheless, unfortunately, so far, most schools' common reporting practices in math tests are still based on classical test theories, which means that each student can only be provided with a total score. Although this can serve the purpose of ranking students, selecting candidates for projects, etc., it should be emphasized that the design of these assessments cannot naturally provide students with more refined information about their mastery (de la Torre and Minchen, 2014). Therefore, after the test, many teachers can only lead all students to review almost all the knowledge involved in the test and cannot give targeted intervention according to each student's knowledge state. This not only limits the progress of students but also limits teachers to reflect on and adjust their own teaching methods and content. In recent decades, researchers have proposed the cognitive diagnostic theory to solve the above problems (Gentile et al., 1969; Embretson, 1998; Tatsuoka, 2009; Wu, 2019).

As a product of the combination of psychometrics and cognitive psychology, the cognitive diagnostic theory is considered the new generation of measurement theory. Specifically, the cognitive diagnosis is a modeling approach aimed at providing examinees' fine-grained information on unobservable (i.e., latent) attributes required to solve specific items (Templin and Bradshaw, 2013). These attributes refer to the knowledge, skills, strategies, etc., which describe mental processing when solving the problem (Nichols et al., 1995; Chen et al., 2012). We can analyze the students' strengths and weaknesses in specific learning fields to remedy students' learning and improve teaching quality based on such fine-grained information.

Researchers have carried out some research in statistics or mathematics tests in middle schools based on cognitive diagnostic theory. For example, Chen (2012) produced a diagnostic description of urban and rural students' cognitive knowledge, abilities, and skills related to TIMSS 1999 mathematics items in Taiwan; Arican and Sen (2015) analyzed the differences between Turkish and South Korean eighth-grade students in attributes involved in the TIMSS 2011 mathematics test and analyzed Turkish students' strengths and weaknesses on these attributes; Lee et al. (2011) used the data of TIMSS 2007 fourth grade mathematics test to show that when using a specific cognitive diagnostic model, there is an incredible wealth of fine-grained information that can be translated directly for classroom application at the attribute level.

Although researchers have conducted some studies with cognitive diagnosis in mathematical tests, unfortunately, most of these studies (1) are based on large-scale assessments with a large number of participants and many items covering a wide range of content. Therefore, these studies may be used to provide references for the macropolicy formulation, such as modifying the focus of the curriculum in subsequent years, but they may not be able to promote immediate changes in the teaching of specific knowledge in a particular classroom; (2) use existing tests in the analysis, which are not guided by cognitive diagnosis when they are developed; (3) usually only use cognitive diagnosis to analyze the test data but do not use the analysis results to carry out targeted interventions on students, nor do they investigate the effects of the interventions (Lee et al., 2011; Chen, 2012).

To sum up, based on the class's actual teaching process, this study selected a specific topic of junior high school mathematics curriculum in China, specifically, the characteristics of the data distribution as the research content, and used the cognitive diagnosis to analyze the students' knowledge mastery. Then, guided by the analysis results, this study carried out targeted interventions and verified the intervention effects. This research's primary purpose is to answer the following two questions in the context of actual class tests: (1) how do students master each attribute involved in the data distribution characteristics and (2) whether the results of cognitive diagnosis can be used to intervene with students so as to improve their mastery of attributes effectively. By answering these two questions, this study not only provides an example for in-service educators to conduct cognitive diagnosis but also provides some suggestions for classroom teaching on the data distribution characteristics at both the classroom and individual levels. Besides, following the cognitive diagnosis process, this study developed two sets of instruments for measuring the knowledge about the data distribution characteristics, which were used for pre- and post-tests (the details will be mentioned later).

## Materials and Methods

### Participants

In this study, 105 eighth-grade students in two parallel classes taught by the same mathematics teacher participated with no payment offered. Among them, 52 students in the first class were in the experimental condition, and 55 students in the other class were in the control condition. In addition, three students in the experimental group and two students in the control group failed to participate on the test day. Therefore, in the end, there were 49 people in the experimental condition and 53 people in the control condition. Gender distribution was as follows: 43 boys (42%) and 59 girls (58%) in total; 18 boys (37%) and 31 girls (63%) in the experimental group; and 25 boys (47%) and 28 girls (53%) in the control group.

It should be noted that although the students in the experimental and control groups were directly grouped by class, these students still had similar ability levels because of the school's class placement policy. Moreover, all participants were taught by the same mathematics teacher, making it more likely that students in these two classes have the same or similar attribute mastery.

The participants were from an ordinary junior high school in a central province of China. The educational situation in this region is a good representative of the overall educational situation in China.

### Q-Matrix and Model

In general, in order to conduct cognitive diagnosis analysis, the preliminary work is to determine the Q-matrix and select the appropriate cognitive diagnosis model (CDM).

The Q-matrix is a binary matrix whose elements only take 0 or 1. In cognitive diagnosis, it represents the relations between the test items and attributes; that is, it indicates which attributes are required to correctly answer each item (Tatsuoka, 1983, 1985; Henson et al., 2007; Ravand, 2016). Therefore, the Q-matrix can guide the development of instruments from the perspective of cognitive psychology, making it play an essential role in cognitive diagnosis (Leighton and Gierl, 2007). The columns in the Q-matrix represent attributes, and the rows represent items. Given *J* items and *K* attributes, if the attribute *k* (*k* = 1, 2, …, *K*) is measured in item *j* (*j* = 1, 2, …, *J*), then the element *q*_*jk*_ = 1, otherwise *q*_*jk*_ = 0. It should be noted that when constructing the Q-matrix, the hierarchical structure between attributes should be considered (Gierl et al., 2007). In the following instrument development, the Q-matrix determined by this research will be presented in detail.

In terms of CDMs, researchers have proposed a large number of models in the past few decades. According to different assumptions about the relationship between attributes, these CDMs can be roughly divided into three categories: (1) compensatory models, in which participants' lack of specific knowledge required for correct answers can be compensated by other knowledge they mastered, such as linear logistic test model (LLTM; Fischer, 1973) and deterministic inputs, noisy “or” gate model (DINO; Templin and Henson, 2006); (2) non-compensatory models, in which participants' lack of knowledge required for correct answers cannot be compensated by other knowledge, such as rule space method (RSM; Tatsuoka, 1983, 2009) and deterministic inputs, noisy “and” gate model (DINA; Haertel, 1989; Junker and Sijtsma, 2001); and (3) general models, that is, models that can be converted into compensated or non-compensated models after adding constraints, such as general diagnostic model (GDM; von Davier, 2008) and log-linear CDM (LCDM; Henson et al., 2009).

Among these models, the non-compensatory models may be more appropriate for mathematical tests because all steps must be successfully answered when solving a mathematical problem, which is consistent with the assumption of the non-compensatory models (Chen, 2012). Although theoretically, the non-compensatory models are more suitable for the content of this study, in the analysis process, we still considered both the classic non-compensatory models and the compensatory models, specifically DINA, DINO, and generalized DINA (G-DINA; de la Torre, 2011), and compared their fitness.

### Design and Procedure

This study followed an experimental design to verify the effectiveness of the DINA model's analysis results and the analysis-based interventions in the context of the actual class test, that is, a small number of participants in specific classes and a small number of items with specific content. The study was divided into three stages: pre-test, intervention, and post-test. The pre- and post-tests were the same for the two groups, but the intervention was different.

During the intervention, the experimental group was asked to read the one-page targeted intervention materials, which included explanations of their poorly mastered knowledge and corresponding exercises. The control group was asked to read the non-targeted intervention materials that contained all the knowledge involved in the tests. In all reading materials, the exercises were accompanied by ideas and answers. Students in both groups had 2 days to read their materials on their own.

Before and after the intervention (also called experimental treatment), pre- and post-tests were applied, respectively. Each test had a proximate duration of 25 min to respond to 17 items. Moreover, the two tests were exactly the same in terms of item format, and the items in the corresponding positions measured the same attributes. In addition, since the composition of the experimental group's intervention materials was determined by the analysis results of the DINA model, the interventions of the experimental and the control groups started on the day after the pre-test.

According to the Helsinki Declaration (World Medical Association, 2013), we strictly followed the ethical principles for psychological research. We informed all the participants of this study's purpose and ensured that they all understood our purpose and the possible benefits of proper participation. It was possible to drop out of the study, but no participant dropped out.

### Instruments Development

In this study, following the development process of cognitive diagnosis tests described below, two sets of instruments for measuring the knowledge about data distribution characteristics were developed, which were used for pre- and post-tests.

#### The Attributes of Data Distribution Characteristics and Their Hierarchical Structure

The quality of the diagnostic assessment is affected by how correctly the attributes underlying the items of any given test have been specified (Ravand, 2016). A variety of sources can be used to define attributes involved in a test, such as test specifications, analysis of item content, think-aloud protocol, and the results obtained from related research (Leighton et al., 2004; Leighton and Gierl, 2007).

Through studying the Chinese Mathematics Curriculum Standards for Full-Time Compulsory Education, the think-aloud protocol, and consulting experts, researchers determined the six main attributes in the characteristics of the data distribution and the hierarchies of these attributes. The six attributes were sort (A1), median (A2), average (A3), variance (A4), weighted average (A5), and mode (A6).

Then, the hierarchical structure between the above attributes was preliminarily analyzed as follows.

First, consider the relationship between sort, average, and median. For a set of data with odd numbers, as long as the students master the concepts of sort and median, they can find the median. However, for a set of data with even numbers, students also need to calculate the average of the two numbers in the middle. That is, they need to master the concept of average. Therefore, the sort and average are the direct prerequisites for the median.

Second, the average is a direct prerequisite for the variance. Since the average is involved in the calculation of variance, the average is a prerequisite for the variance. Moreover, there is no such attribute. While it is a prerequisite for variance, the average is its prerequisite. Therefore, the average is a direct prerequisite for the variance.

Next, the average is also a direct prerequisite for the weighted average. Only by mastering the average can students master the weighted average, so the average is a prerequisite for the weighted average. Furthermore, there is no such attribute. While it is a prerequisite for the weighted average, the average is its prerequisite. Thus, the average is said to be the direct prerequisite for the weighted average.

Lastly, the mode is not related to other attributes. For a particular set of data, students can find the mode of this set by simple counting. On the other hand, if students do not master the concept of mode, they may still master other attributes. For this reason, the mode is independent of other attributes.

Then, we selected six students who were also in the eighth grade to conduct think aloud. Then, the relationship between these attributes was further analyzed through this method. This process included three steps: training, formal experiment, and analysis. First, the researcher explained the specific requirements for thinking aloud and took three specific items as examples to describe the thinking process in solving the problems. After the students fully understood this process, we asked each student to use the think-aloud method to report on the process of solving 10 items about the data distribution characteristics. Finally, the students' reports were summarized and analyzed. Based on the above analysis and the opinions provided by the think-aloud protocol and experts, it can be considered that the hierarchical structure of these attributes is as follows.

#### Q-Matrix and Instrument Development

**Figure 1:**
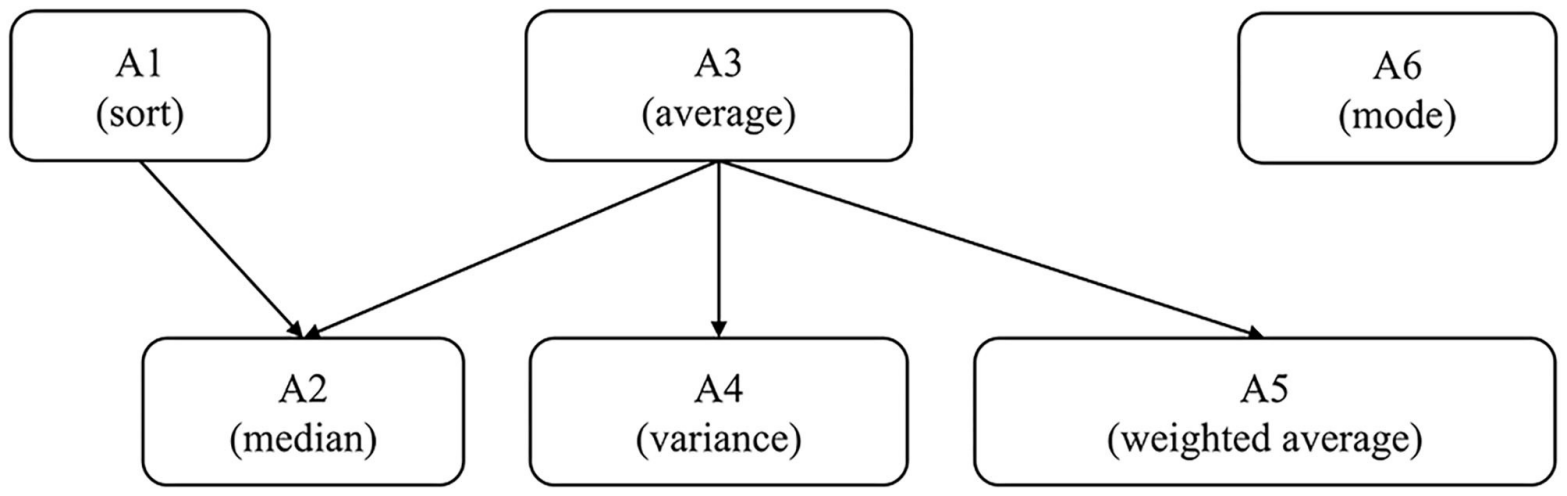
The hierarchical structure of the six attributes of this study.

Based on the hierarchies, we can obtain the reachability matrix, R, which reflects the direct and indirect connections between attributes. If the attribute *k* is reachable from attribute *k*′, that is, attribute *k*′ is a prerequisite of attribute *k*, then *r*_*k*^′^*k*_, otherwise, *r*_*k*^′^*k*_. Therefore, the reachability matrix, R, corresponding to Figure 1 is,

$$\begin{array}{l} R=\left(\begin{array}{cccccc} 1 & 1 & 0 & 0 & 0 & 0\\ 0 & 1 & 0 & 0 & 0 & 0\\ 0 & 1 & 1 & 1 & 1 & 0\\ 0 & 0 & 0 & 1 & 0 & 0\\ 0 & 0 & 0 & 0 & 1 & 0\\ 0 & 0 & 0 & 0 & 0 & 1 \end{array}\right). \end{array}$$

The next step was to construct a set of potential items. In the case of the six attributes in this study, if the hierarchical structure is not considered, then the size of this set is related to the number of attributes *K* (i.e., 2^6^−1 = 63). As mentioned above, the Q-matrix can be used to display such a set of potential items. However, if the attribute hierarchy is taken into consideration, how to obtain a set of potential problems that can satisfy these hierarchies? Ding et al. (2009) proposed that starting from the R matrix and using the expansion algorithm, a set of potential items that conforms to the hierarchical structure can be efficiently obtained. Based on their method, this research obtained a set of all the potential items that satisfy the hierarchies, denoted as initial Q-matrix, as shown in Table 1 (if a row with all 0s was added, it can also represent all possible knowledge states of students).

**Table 1:** Initial Q-matrix.

**Items**	**A1**	**A2**	**A3**	**A4**	**A5**	**A6**
1	1	0	0	0	0	0
2	0	0	1	0	0	0
3	1	0	1	0	0	0
4	1	1	1	0	0	0
5	0	0	1	1	0	0
6	1	0	1	1	0	0
7	1	1	1	1	0	0
8	0	0	1	0	1	0
9	1	0	1	0	1	0
10	1	1	1	0	1	0
11	0	0	1	1	1	0
12	1	0	1	1	1	0
13	1	1	1	1	1	0
14	0	0	0	0	0	1
15	1	0	0	0	0	1
16	0	0	1	0	0	1
17	1	0	1	0	0	1
18	1	1	1	0	0	1
19	0	0	1	1	0	1
20	1	0	1	1	0	1
21	1	1	1	1	0	1
22	0	0	1	0	1	1
23	1	0	1	0	1	1
24	1	1	1	0	1	1
25	0	0	1	1	1	1
26	1	0	1	1	1	1
27	1	1	1	1	1	1

It should be pointed out that 10 items were removed from the initial Q-matrix for the following three reasons. First, this study's sample size is relatively small, so if 27 items are all used, it may bring challenges to model estimation. Second, in practice, the attribute A1 (sort) is straightforward to be mastered by middle school students, and it is often examined together with the median in tests. Simultaneously, there are many (66.667%) items related to the sort in the initial Q-matrix. Therefore, we removed a part of those items that examined the sort without examining the median at the same time. Third, for a daily class test, it is unlikely to contain so many items. Therefore, based on experts' and teachers' opinions, we selected 17 typical items from the initial Q-matrix to form the final Q-matrix (Table 2).

**Table 2:** Final Q-matrix.

**Items**	**A1**	**A2**	**A3**	**A4**	**A5**	**A6**
1	1	0	0	0	0	0
2	1	1	1	0	0	0
3	0	0	1	0	0	0
4	0	0	1	1	0	0
5	0	0	1	0	1	0
6	0	0	0	0	0	1
7	1	1	1	0	0	1
8	1	1	1	1	0	0
9	1	1	1	0	1	0
10	0	0	1	1	0	1
11	1	0	1	0	0	1
12	0	0	1	1	1	0
13	1	1	1	1	0	1
14	0	0	1	0	1	1
15	1	1	1	0	1	1
16	1	1	1	1	1	1
17	1	0	1	0	0	0

Finally, this research developed the tests based on the final Q-matrix. For example, according to the third row in the final Q-matrix, the third item should only measure the attribute A3 (i.e., the average). Therefore, researchers compiled an item that only measured the attribute A3, as shown in Table 3.

**Table 3:** Item example.

**Item**	**Item content**
Item 3	If the average of a set of data a, b, c, d is M, then the average of another set of data 2a + 2, 2b + 2, 2c + 2, 2d + 2 is (). A. 2M B. 2M + 1 C. 2M + 1.5 D. 2M + 2

It is easy to know that this item only measures the attribute A3, which is consistent with the Q-matrix. According to this process, the pre- and post-test papers, each containing 17 items, were compiled. All items are multiple-choice items with four response options, and all items are 0–1 scored, with a full score of 17.

### Data Analysis

We conducted descriptive and inferential analyses of the students' responses. Specifically, the analysis of variance (ANOVA) was used to examine the difference in average scores between both the groups (experimental and control group) and the moments (pre- and post-tests). We also used three CDMs mentioned in *Q-Matrix and Model* to analyze the students' responses and chose the most suitable CDM. It is then used for item analysis and used to obtain the attributes of poor mastery of students and students' probability of belonging to each knowledge state. In addition, the reliability of the model was examined. In addition, it should be pointed out that the modeling with the selected CDM was carried out according to the moment. In other words, for the selected model, a total of two modeling was performed, and then, the person parameter estimates were segmented by the condition. There are no missing values. All the data analysis processes were completed in R, and the CDM package was used.

## Results

### Difference Analyses of the Tests Before and After the Intervention

Table 4 shows descriptive analyses of the tests in experimental and control conditions before and after the intervention and the difference between these two moments.

**Table 4:** Means and standard deviations of pre-test, post-test, and the difference between tests in both groups.

	**Pre-test**		**Post-test**		**Pre–post**	
**Group**	**M**	**SD**	**M**	**SD**	**M**	**SD**
Experimental group	12.878	2.395	11.918	3.347	0.960	3.409
Control group	12.906	2.452	9.717	3.559	3.189	3.258

**Figure 2:**
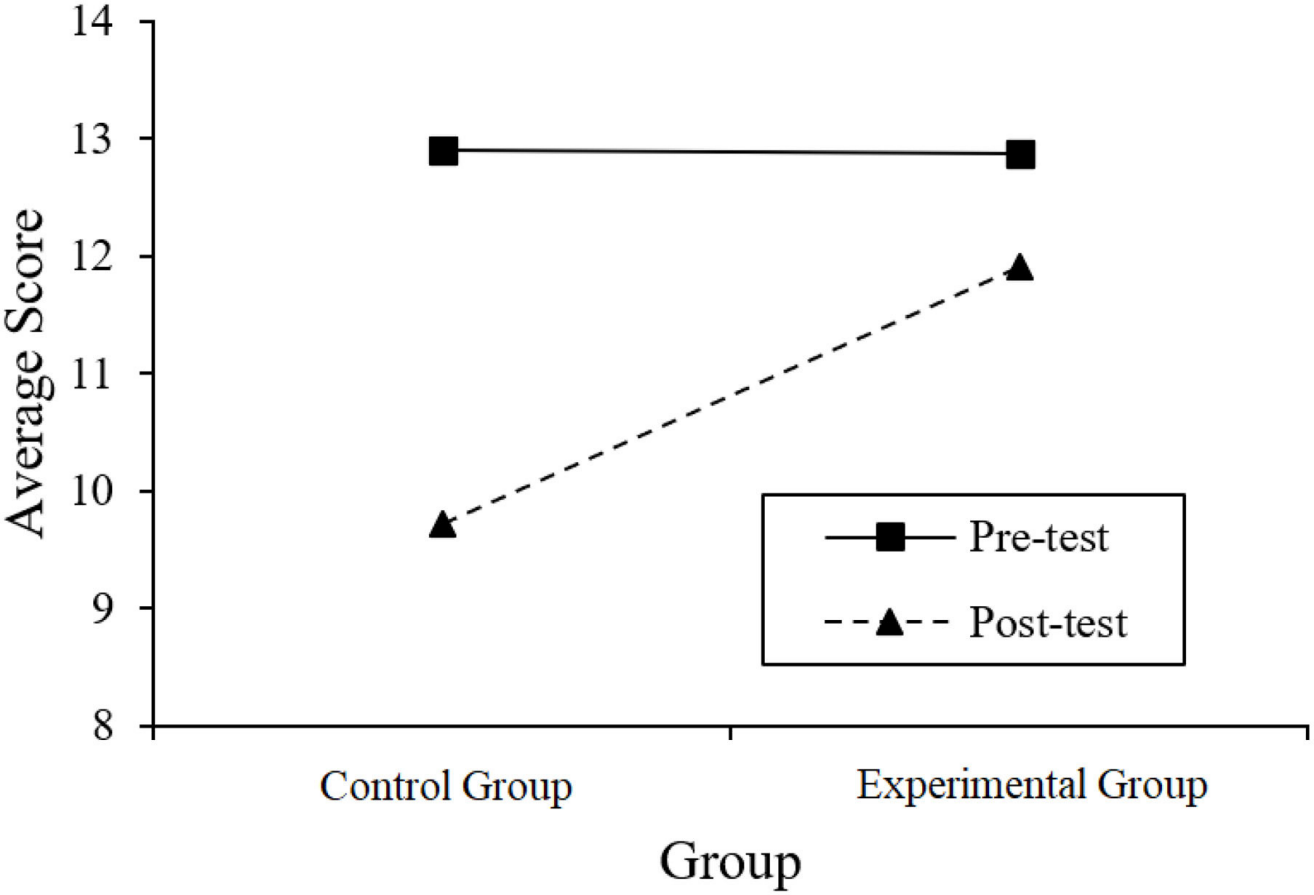
The illustration of time × group interaction.

In order to further clarify whether the difference between average scores in Table 4 is statistically significant, we took an ANOVA with group (experimental and control) as an intersubject variable and time (pre- and post-tests) as a within-subject variable. In addition, Figure 2 shows the interaction between time and groups in the ANOVA.

The results of the ANOVA show that the time × group interaction is significant (*F* = 11.401, *p* = 0.001, *partial η*^2^ = 0.102). This shows that from the pre- to post-test, the difference between the experimental group and the control group has changed. Combining with Figure 2, we can know that (1) the experimental and control groups did not show significant differences in the average scores of the pre-test (*b* = −0.028, *t* = −0.059, *p* = 0.953). To some extent, this confirms our previous assumption that the knowledge mastery level of the two groups of students should be basically the same (because the two groups of students came from two parallel classes). (2) The experimental and control groups showed significant differences in the average scores of the post-test (*b* = 2.201, *t* = 3.211, *p* = 0.001). In addition, it should be pointed out that students' average score in the post-test is lower than that in the pre-test, mainly because the items in the post-test are more difficult. This study took into account that the practice effect may cause the overall students' post-test scores to be too high, making it impossible to distinguish the difference between the experimental group and the control group. Therefore, although the pre- and post-test papers of this study were developed according to the same Q-matrix, the post-test was more difficult.

### Diagnosis Results of the Pre-test and Post-test of the Two Groups

The following shows the fit of the three CDMs to the pre-test data, and the most suitable model is selected for subsequent analysis. Then, some reliability indicators of the model are shown. Last but not the least, it presents the pre-test and post-test diagnosis results of the students in two groups, which is an attribute-aspect analysis of the pre- and post-test of the two groups.

#### Model Fit and Item Analysis

As mentioned earlier, we considered three CDMs (DINA, DINO, and G-DINA). DINA and DINO are conjunctive and disjunctive models, respectively, while G-DINA is a general model that combines DINA and DINO (Sorrel et al., 2016). By evaluating each model's absolute and relative fit, the most suitable CDM can be selected (Sessoms and Henson, 2018). Considering that the DINA and DINO models are nested in the G-DINA model, the DINA and DINO models will always have a lower log likelihood (de la Torre, 2011). Therefore, the likelihood ratio (LR) test can be used to assess whether the observed difference in model fit is statistically significant. If LR is significantly different from 0, the general model fits the data significantly better than the simplified model. In addition, we present the Bayesian information criterion (BIC) of the model, which can also measure the model fit. The smaller the BIC, the better the model fit. Regarding the absolute fit, we used the method proposed by Chen et al. (2012), which is to evaluate the absolute fit. If the evaluated model fits the data well, the maximum χ^2^ statistics should not be zero significantly different.

Table 5 shows the relative fit and absolute fit indices calculated for the G-DINA, DINA, and DINO models. Among them, the BIC of the DINA model is the smallest. The two LR tests, respectively corresponding to the comparison of the G-DINA model with the DINA (*LR* = 124.572) and DINO (*LR* = 165.026) models, are not significant (*p*>0.05), which shows that the more parsimonious models (DINA and DINO) do not result in significant loss of fitting. Absolute item fit statistics also indicated that the DINA model has a better fit than the other models. For all three models, the maximum χ^2^ statistics were not significant at a-level of 0.05 after applying the Holm–Bonferroni correction (Holm, 1979). In short, the DINA model fits the pre-test best, which is also consistent with the theoretical analysis mentioned in *Q-Matrix and Model*. Thus, the DINO and G-DINA models are discarded, and the DINA model is further examined for its adequacy to model the post-test.

**Table 5:** Model fit indices for different cognitive diagnosis models.

				**LR test**			**Absolute fit**		
**Model**	**Log-like**	**BIC**	**Np**	**LR**	***df***	***p***	**abs(fcor)**	**max(**χ**^2^)**	***p***
G-DINA	−671.300	2527	256				0.251	8.148	0.586
DINA	−733.586	1749.295	61	124.572	195	0.999	0.271	5.413	1.000
DINO	−753.813	1789.749	61	165.026	195	0.942	0.374	12.109	0.068

*Np is the number of parameters*.

In addition, we also need to confirm whether the hierarchical structure defined in Figure 1 and the final Q-matrix defined in Table 2 are correct. To test the correctness of the hierarchy, we modeled the pre-test using a saturated DINA model without considering the hierarchical structure. The results show that the BIC of the saturated model is 1915.794, which is slightly larger than the simple model. According to the study of Akbay and de la Torre (2020), this means that the structure is correct because the simpler model considering the hierarchical structure has a similar fit to the saturated model where the probability for all the possible latent classes is estimated (i.e., 2^6^). For the Q-matrix, according to the research of Sorrel et al. (2016), the modification of the Q-matrix should make theoretical sense. For the test instruments developed by this research, it is very clear whether each item has measured specific attributes. Therefore, although a few rows of the Q-matrix are suggested to be modified according to the methods proposed by Chiu (2013) and de la Torre (2008), we did not adjust the final Q-matrix.

Finally, the reliability of CDM scores was tested. According to the pre- and post-test data, the classification accuracy and consistency proposed by Cui et al. (2012) are calculated based on simulation, as shown in Table 6. It can be seen that the classification accuracy and consistency of the student pattern (i.e., the student's knowledge states) are >0.75, and the classification accuracy and consistency of the attributes are >0.9, except for a few cases.

**Table 6:** Classification accuracy and consistency.

			**Attributes**					
**Time**	**Indicator**	**Pattern**	**A1**	**A2**	**A3**	**A4**	**A5**	**A6**
Pre-test	Accuracy	0.765	0.961	0.917	0.990	0.931	0.833	0.980
	Consistency	0.784	0.980	0.931	0.980	0.902	0.824	0.961
Post-test	Accuracy	0.817	0.931	0.906	0.960	0.936	0.941	0.941
	Consistency	0.723	0.881	0.832	0.941	0.891	0.901	0.901

So far, it can be considered that by modeling the pre- and post-test through the DINA model, we can obtain accurate diagnosis results. The following are analyses of the items and the attributes. Table 7 shows some information about the pre- and post-test at the item level. It can be seen that although some guess parameters are relatively large, the guessing parameters and slipping parameters for most items are within a reasonable range. As mentioned earlier, the tests are 0–1 scored, and no points are deducted for wrong answers, which can partly explain the larger guessing parameters. Simultaneously, due to the small sample size, these estimates' standard deviation may be large, which brings some challenges to interpreting those extreme item parameter estimates. These explanations need to be given in conjunction with the specific item content, and interested readers can contact us for the item content. In addition, according to the standard of Kunina-Habenicht et al. (2009, 2012), 76.471% of the items in the pre-test show moderate or good fit [root mean square error approximation (RMSEA) <0.1], and the proportion in the post-test is 82.353%.

**Table 7:** Item information.

	**Pre-test**					**Post-test**			
**Item**	**P_c_**	**g**	**s**	**RMSEA**	**Item**	**P_c_**	**g**	**s**	**RMSEA**
1	0.990	1.000	0.010	0.036	1	0.961	0.878	0.000	0.087
2	0.882	0.769	0.094	0.066	2	0.814	0.746	0.143	0.083
3	0.843	0.000	0.104	0.038	3	0.765	0.214	0.125	0.072
4	0.569	0.185	0.182	0.060	4	0.627	0.002	0.188	0.060
5	0.941	0.856	0.000	0.046	5	0.588	0.252	0.263	0.076
6	0.971	0.751	0.011	0.061	6	0.686	0.344	0.169	0.077
7	0.824	0.269	0.063	0.054	7	0.725	0.409	0.000	0.067
8	0.637	0.374	0.169	0.087	8	0.608	0.315	0.166	0.085
9	0.912	0.830	0.000	0.068	9	0.333	0.124	0.485	0.080
10	0.676	0.218	0.000	0.053	10	0.696	0.452	0.172	0.102
11	0.745	0.000	0.191	0.054	11	0.735	0.401	0.045	0.099
12	0.500	0.259	0.260	0.126	12	0.627	0.406	0.253	0.099
13	0.696	0.730	0.329	0.126	13	0.529	0.382	0.318	0.207
14	0.980	0.954	0.000	0.041	14	0.627	0.108	0.142	0.068
15	0.363	0.267	0.534	0.111	15	0.490	0.272	0.321	0.107
16	0.539	0.463	0.376	0.187	16	0.343	0.115	0.421	0.070
17	0.824	0.509	0.150	0.053	17	0.618	0.289	0.226	0.094

*P_c_ is the proportion of correct; g and s are the item parameters involved in the DINA model, representing the probabilities of slipping and guessing, respectively; RMSEA is the root mean square error approximation*.

#### The Probabilities of Students' Mastery of the Attributes

Table 8 shows the probabilities of students' mastery of the attributes. In the pre-test, the students' mastery of the attributes is generally good, and the probabilities of students' mastery of the attributes show consistent characteristics in both groups. Specifically, students had a good grasp of A1, A2, A3, and A6 (the probabilities are above 0.7) but had a poor grasp of A4 and A5 (the probabilities are around 0.6).

**Table 8:** The probabilities of students' mastery of the attributes in both conditions and moments.

	**Pre-test**		**Post-test**	
**Attributes**	**Experimental group**	**Control group**	**Experimental group**	**Control group**
A1	1.000	0.962	0.837	0.566
A2	0.857	0.830	0.776	0.566
A3	0.959	0.925	0.918	0.717
A4	0.551	0.717	0.878	0.679
A5	0.653	0.679	0.837	0.585
A6	0.959	0.887	0.837	0.585

The intervention materials mentioned above were developed based on the results of this analysis. For the experimental group, the one-page targeted intervention materials they read only include the explanation of A4 and A5 and the corresponding exercises, while the materials read by the control group involve all the six attributes, from A1 to A6.

In the post-test, compared with the control group, the probabilities of experimental group show that the targeted intervention was successful. Specifically, in the experimental group, the probability of students' mastery of A4 was increased from 0.551 to 0.878 (χ^2^ = 12.800, *p* = 0.000), and the probability of students' mastery of A5 was raised from 0.653 to 0.837 (χ^2^ = 4.350, *p* = 0.037). However, in the control group, the probability of students' mastery of A4 was changed from 0.717 and 0.679 to 0.679 and 0.585 (χ^2^ = 0.179, *p* = 0.672;χ^2^ = 1.014, *p* = 0.314), respectively. It indicates that from the pre-test to the post-test, the probabilities of the experimental group students' mastery of the two attributes, A4 and A5, were increased obviously. However, for the control group, the changes in the probabilities were fluctuated and relatively smaller. What is more, the changes in the control group were not significant. Therefore, it shows that the post-test result of the control group, especially the students' mastery of A4 and A5, is not as good as the experimental group.

#### The Probability of Students Belonging to Each Possible Knowledge State

Table 9 shows the probability of students belonging to each possible knowledge state, which can be seen as a more specific expansion of Table 8. In the pre-test, although there are some differences in the probabilities between the experimental group and the control group, the knowledge states with higher probability all correspond to not mastering A4 or A5 or both of them. The results of the post-test show that except for the knowledge state of 111111, the probability of the experimental group students belonging to any other knowledge state is <0.1, which indicates that the students in the experimental group do not have a particularly poor grasp of any attribute, but the students in the control group still have poor grasp of certain attributes.

**Table 9:** The probability of students belonging to each possible knowledge state in both conditions and moments.

	**Pre-test**		**Post-test**	
**Knowledge states**	**Experimental group**	**Control group**	**Experimental group**	**Control group**
000000	–	–	0.082	**0.283**
100000	0.041	0.075	–	–
001000	–	–	–	–
101000	–	–	–	–
111000	–	–	–	–
001100	–	–	0.020	0.038
101100	–	–	–	–
111100	–	–	0.061	0.094
001010	–	–	–	–
101010	–	–	–	–
111010	–	–	–	–
001110	–	0.038	–	–
101110	–	–	–	–
111110	–	–	–	–
000001	–	–	–	–
100001	–	–	–	–
001001	–	–	–	–
101001	–	–	–	–
111001	**0.306**	**0.170**	–	–
001101	–	–	–	–
101101	–	–	–	–
111101	–	0.075	–	–
001011	–	–	–	–
101011	**0.102**	0.038	–	–
111011	–	–	0.041	0.038
001111	–	–	0.061	**0.113**
101111	–	0.019	0.061	–
111111	**0.551**	**0.585**	**0.673**	**0.434**

*In bold are the probabilities >0.1. The dashes indicate that these probabilities are <0.001*.

## Discussion

Through cognitive diagnosis, this study analyzed the junior high school students' mastery of the six attributes involved in the data distribution characteristics and used the analysis results to conduct targeted interventions on the students in the experimental group. The results show that among the attributes, students had a relatively poor grasp of A4 (variance) and A5 (weighted average) before the intervention. After the intervention, compared with those who read the non-targeted material, those who read the targeted material significantly improved their mastery of the variance and the weighted average. These results answer the two questions raised by this research very well.

On the one hand, the pre-test results in Table 5 directly indicate that the probability of students mastering the attributes A4 and A5 is low. At the same time, the analysis of the students' knowledge states in Table 6 shows that before the intervention, students have a higher probability of belonging to the following knowledge states: 111111, 111001, and 101011. It is consistent with the analysis of attributes in Table 5 because most of these states indicate that attribute variance or weighted average is not mastered. In many previous studies, the weighted average was also regarded as a difficulty (Pollatsek et al., 1981; Day et al., 2014). However, except for a few studies, the variance is rarely mentioned. In fact, as a measure of the data variation, the variance is also difficult to grasp by students (Koparan, 2015). This is mainly due to the following two reasons: (1) The calculation of variance involves multiple steps such as calculating the average, square, and the sum of polynomials, which makes it easy to make mistakes. (2) The concept of variance is abstract (Sinitsky and Ilany, 2009). As for the weighted average, it is calculated by averaging after assigning different weights to each data. After communicating with the class's mathematics teacher, it was found that because she believed that the weighted average was an extension of the average, she did not spend much time explaining to the students in detail, which may lead to students' poor understanding of the concept of weight.

On the other hand, the results show that those participants who read the targeted material improved their mastery of the variance and the weighted average. In contrast, those who read the non-targeted material did not experience any significant improvement, which reveals that the targeted reading material is effective. In addition, since the targeted reading material was developed based on the pre-test diagnosis results, the diagnosis results are also valid.

According to the results of this research, some teaching suggestions on data distribution characteristics can also be provided. Here are some suggestions for classroom teaching through the diagnosis results, the intervention, and the communication with teachers. In the teaching of weighted average, it is recommended that teachers should not only teach students its calculation formula but also pay more attention to the explanation of the concept of weight in the weighted average. Considering the weight's abstractness, it is suggested that teachers should actively use examples from life to help students understand the weight. On the one hand, in the teaching of variance, it is necessary to help students understand the concept as intuitively as possible. On the other hand, since the calculation process of variance is relatively complicated, more exercises should be given to students on the variance calculation.

Furthermore, the following are some learning suggestions on the individual level for certain knowledge states. These knowledge states are those whose probabilities are >0.1 in the pre-test or those who have mastered other attributes but not A4 or A5.

(1) The students with a knowledge state of 111011 only have a poor grasp of variance, a difficult-to-understand attribute, indicating that they have sufficient learning ability. Therefore, it is recommended to conduct a targeted review, grasp the basic concept, and calculation method of variance.
(2) The students with a knowledge state of 111001 have a poor grasp of the two attributes of weighted average and variance. These two attributes are the attributes that the entire classes have a poor grasp of. It is recommended that such students review the definitions of weighted average and variance, find examples in life to help understand, and do a certain number of exercises.
(3) The students with a knowledge state of 111101 have a poor grasp of the weighted average. It is recommended that such students start with the concept of weight, grasp the basic concept of weighted average, and do exercises to help themselves master it.
(4) Students with a knowledge status of 101011 have a poor grasp of the median and variance. Compared with other attributes, especially the weighted average, it is not that difficult to grasp the attribute of the median. It should also be noted that the calculation of the median involves judging whether the number of data is odd or even. Therefore, this kind of students may not study hard and may think that they have mastered certain attributes, when in fact they only know a rough idea. It is recommended that they actively adjust their mentality, review the textbooks, and do a certain number of exercises for the median and variance.

This study is not exempt from limitations. A major limitation is that the post-testing items are too difficult. Consequently, the diagnosis results show that in the post-test, the probabilities of students' mastery of certain attributes are lower than in the pre-test. This brings certain difficulties to the interpretation of the results and makes the results unintuitive. In addition, the sample size of this study is indeed small, which brings certain challenges to parameter estimation. Finally, the intervention of this study can be further refined, such as conducting the individual-based intervention.

In general, this study provides an example to show that in the actual class tests that usually have few participants and few items, the cognitive diagnosis can be used to obtain a relatively accurate students' knowledge state. Then, remedial teaching can be developed based on these results. In other words, this study guides in-service educators to use cognitive diagnosis to reflect on their teaching methods, adjust teaching content, and carry out remedial teaching in the teaching process. Finally, based on the CDM, some suggestions for classroom teaching and individual learning on the topic of data distribution characteristics are given.

## Data Availability Statement

The raw data supporting the conclusions of this article will be made available by the authors, without undue reservation.

## Ethics Statement

The studies involving human participants were reviewed and approved by Institutional Review Board of the Faculty of Psychology, BNU. Written informed consent to participate in this study was provided by the participants' legal guardian/next of kin.

## Author Contributions

HR provided original thoughts and completed the writing of this article. NX and YL provided key technical support. SZ and TY participated in all research procedures and gave important guidance. All authors contributed to the article and approved the submitted version.

## Conflict of Interest

The authors declare that the research was conducted in the absence of any commercial or financial relationships that could be construed as a potential conflict of interest.
